# Surgical Repositioning of a Dilacerated Impacted Incisor

**DOI:** 10.5005/jp-journals-10005-1082

**Published:** 2011-04-15

**Authors:** Vivek Kumar Adlakha, Preetika Chandna, Sumir Gandhi, Saroj Chopra, Namita Singh, Shamsher Singh

**Affiliations:** 1 Senior Lecturer, Department of Pedodontics and Preventive Dentistry, Subharti Dental College, Meerut, Uttar Pradesh, India; 2 Reader, Department of Oral Surgery, Christian Dental College, Ludhiana, Punjab, India; 3 Professor, Department of Pedodontics and Preventive Dentistry, Christian Dental College, Ludhiana, Punjab, India; 4 Lecturer, Department of Pedodontics and Preventive Dentistry, Surindra Dental College, Ganganagar, Rajasthan, India

**Keywords:** Dental trauma, Impacted, Dilaceration.

## Abstract

Dilaceration is one of the most common complications of trauma to deciduous dentition. The possible causes of dilaceration are trauma and developmental disturbances, while some authors suggest an association with some developmental syndromes. Dilaceration can be seen in both the permanent and deciduous dentition. The present case report describes surgical repositioning of a dilacerated impacted maxillary central incisor in a 9-year-old boy.

## INTRODUCTION

Dilaceration refers to an angulation or a sharp curve in the root or crown of a formed tooth. Dilaceration is usually associated with dental trauma to a deciduous predecessor in which the tooth is driven apically into the jaw. The anomaly in tooth happens during the period when the tooth is forming. Trauma results in change of the calcified portion of the tooth and the remaining tooth develops at an angle. The curve or bend in the tooth may occur anywhere along the length of the tooth, depending upon the amount of tooth formed when the injury occurred.^1^ The most complicated situation is root dilacerations with the crown in an inverted direction; thus, the tooth is always impacted.^2^ The conventional treatment for a dilacerated incisor is extraction, transplantation or surgical/ orthodontic approach.^2-7^ This article describes a rare case of dilaceration with the crown in an inverted direction with the palatal aspect of the crown facing the labial side.

## CASE REPORT

**Fig. 1:**
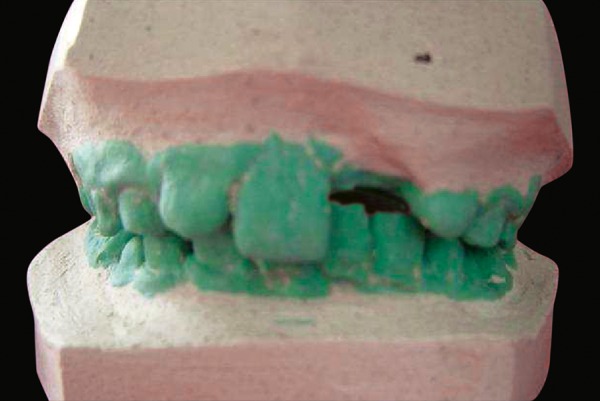
Preoperative study model shows noneruption of tooth number 21

**Fig. 2:**
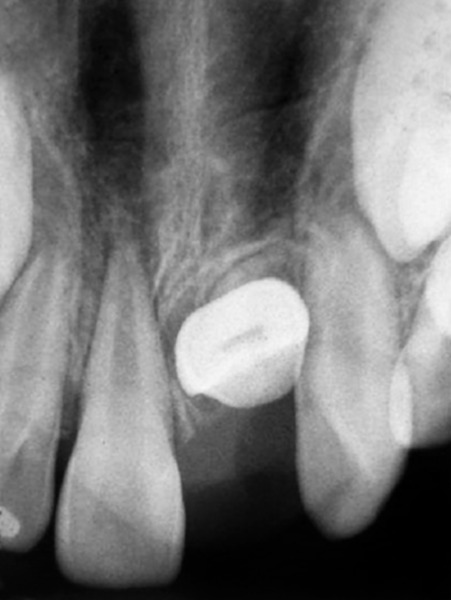
Preoperative IOPA radiograph

**Fig. 3:**
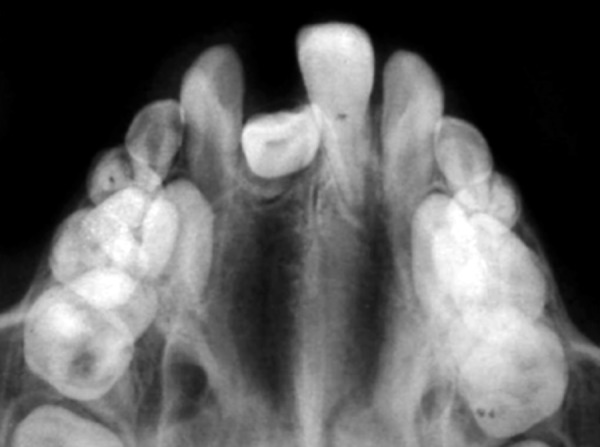
Preoperative occlusal radiograph

**Fig. 4:**
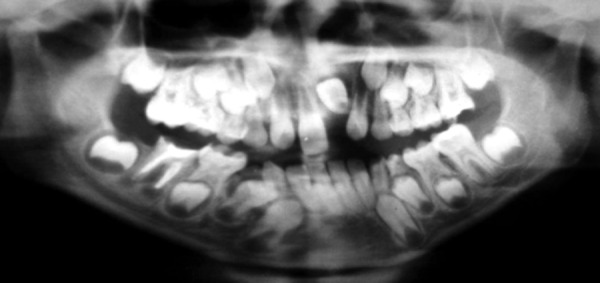
Preoperative OPG radiograph

A 9-year-old boy visited the outpatient clinic of Department of Pedodontics and Preventive Dentistry, Christian Dental College, Ludhiana, India with a chief complaint of missing upper anterior tooth (Fig. 1). Patient gave a history of trauma at the age of 3 to 4 years due to hand pump, which resulted in intrusion of the deciduous maxillary central incisors. The deciduous maxillary central incisors were mobile and were extracted. Intraoral examination revealed missing left maxillary central incisor (tooth no. 21). There was a space deficiency in the maxillary anterior area due to migration of number 11 into the space that would have been occupied by tooth number 21. The maxillary central incisor, with a dilacerated root and the crown directed upwards, was visible on the radiographs (Figs 2 to 4). The treatment goal was to reopen the incisor space and bring the impacted incisor into its position.

## TREATMENT

**Fig. 5:**
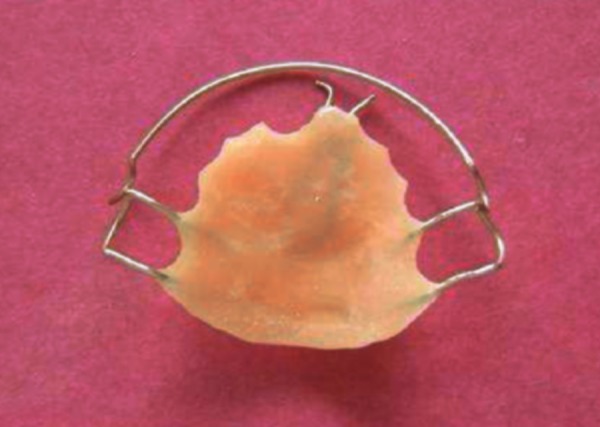
Hawley’s appliance with finger spring for space regaining

**Fig. 6:**
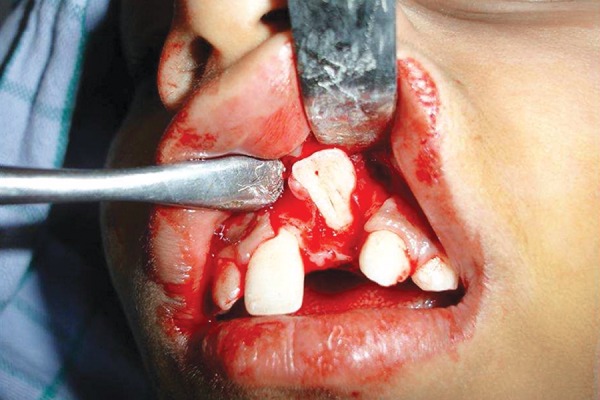
During surgical exposure shows the impacted incisor with its crown inverted

**Fig. 7:**
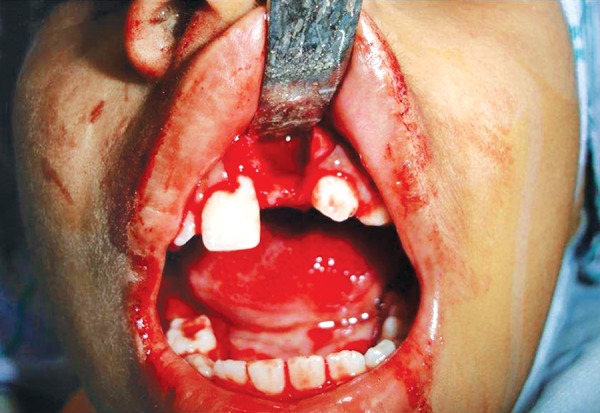
Bony socket after removal of tooth number 21

**Fig. 8:**
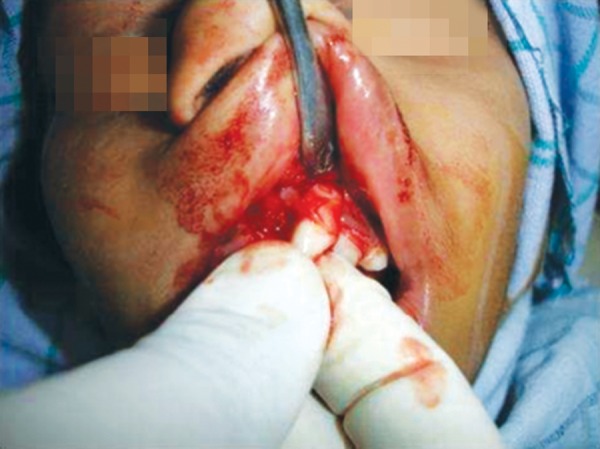
Repositioning of the dilacerated tooth

**Fig. 9:**
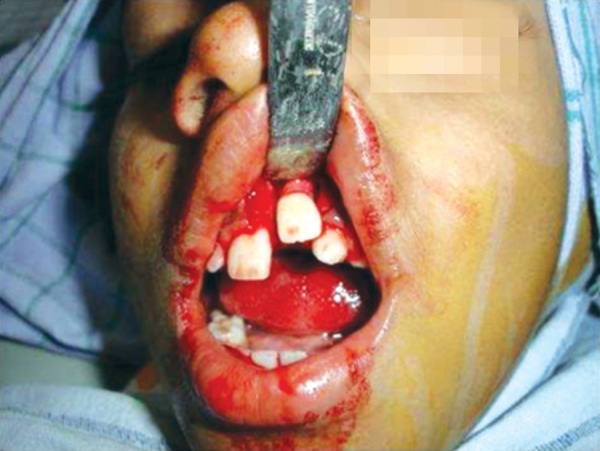
Repositioned tooth number 21 in semierupted state

**Fig. 10:**
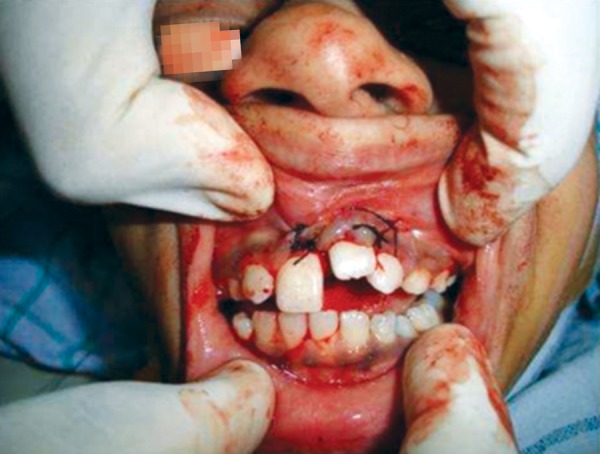
Sutured flap

**Fig. 11:**
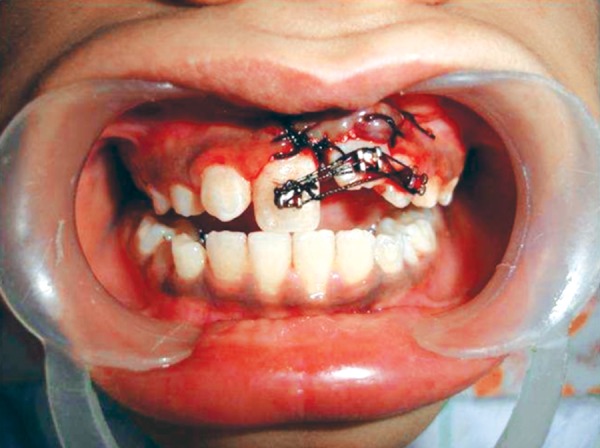
Suture splint in place

**Fig. 12:**
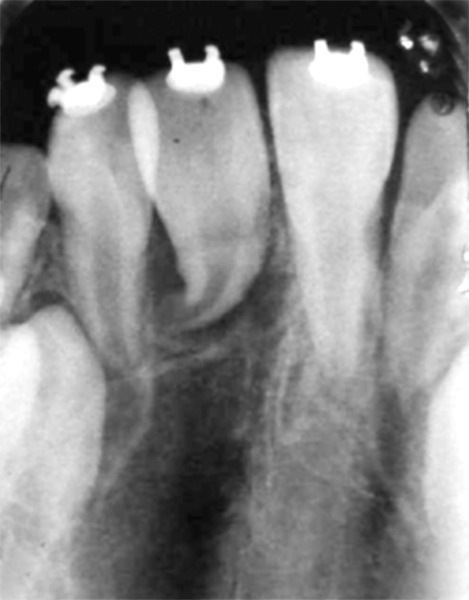
Postoperative IOPA radiograph

Orthodontic treatment was carried out to reopen the lost space using a removable Hawley’s appliance with finger springs (Fig. 5). Once the lost space was regained, surgical repositioning of the dilacerated incisor was planned. Following presurgical investigations, surgical repositioning was undertaken under local anesthesia. The impacted incisor was exposed with a buccal flap operation utilizing vertical releasing incisions. The bone surrounding the crown of the tooth was carefully removed and the incisor tooth follicle was gently separated from its bony socket. Special care was taken not to damage the periodontal membrane on the cervical and root portion of the tooth. The incisor was then repositioned in the correct direction but in the semi-erupted position to safeguard marginal bone regeneration. Orthodontic bracket was cemented on the labial surface of the incisors to splint the repositioned incisor. After suturing the flap, the suture was used to splint the incisor lightly with the adjacent teeth. The sutures and splint were removed after one week (Figs 6 to 12).

**Fig. 13:**
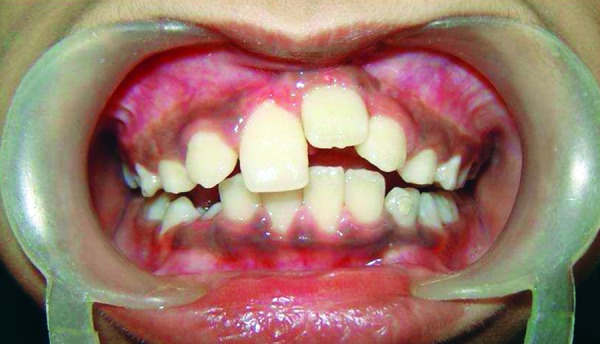
Postoperative photograph after 1 month

The repositioned incisor remained vital and responded normally to percussion, mobility and sensitivity testing. The soft tissue, gingival contour and the probing depth were within normal limits. The patient is presently on orthodontic therapy for further positioning and alignment of the teeth (Fig. 13).

## DISCUSSION

The present case demonstrated that surgical repositioning of a dilacerated impacted maxillary central incisor may be a realistic treatment option as compared to other conventional techniques like extraction, surgical exposure and orthodontic traction, autotransplantation with premolars or autoalloplastic tooth transplantation. It is probably because of the high clinical difficulty in surgicaly positioning the dilacerated impacted incisor tooth; most patients and clinicians probably choose extraction with replacement of prosthesis instead. The present case revealed that dilacerations of the root would not be a great obstacle always if the case had carefully planned procedure.

The various advantages of surgically repositioning the dilacerated impacted incisor are:^2,8^

1. There is only one surgical site and procedure is done once.
2. Simple orthodontic mechanics; if needed.
3. Impacted tooth used as a donor has a thick periodontal membrane, which is more suitable for reattachment than the narrow periodontal membrane found in erupted, functional tooth.
4. Immediate esthetics: Endodontic treatment was not necessary in this case because of the open apex. There is a good chance of revascularization of replanted teeth with open apex unlike those with a closed apex, where endodontic treatment is planned within a couple of weeks after transplantation.^5,9^

Andreasen showed that if autotransplantation was carefully done, success rate can be as high as 95%.^10^

The impacted tooth was transplanted in a semierupted position; immediate esthetics was achieved. This can be a good compliance incentive for both clinicians and parents.

Surgical repositioning treatment helps in achieving normal periodontal and gingival attachment, and normal dental and skeletal growth.

The treatment expense is reduced since the tooth is positioned in a semierupted state. Also, simple mechanics are needed to align the tooth in proper occlusion.^2^ And positioning the tooth in semierupted state helps in safeguarding marginal bone regeneration.^11^

The major disadvantage with surgical repositioning or autotransplantation is injury to the periodontal ligament, resulting in root resorption and/or ankylosis, and injury to the Hertwig’s epithelial root sheath causing partial or total arrest of root development.^10-12^

## CONCLUSION

Surgical repositioning is a viable treatment option for dilacerated impacted incisor. It is a simple surgical procedure with minimal orthodontic mechanics and can be undertaken safely as an outpatient procedure.

## References

[1] Shafer WG., Maynard KH., Bernet ML (1993). A textbook of oral pathology..

[2] Tsai TP (2002). Surgical repositioning of an impacted dilacerated incisor in mixed dentition.. J Am Dent Assoc.

[3] Agnihotri A, Marwah N, Dutta S (2006). Dilacerated unerupted central incisor: A case report.. J Indian Soc Pedod Prev Dent.

[4] Crawford LB (1997). Impacted maxillary central incisor in mixed dentition treatment.. Am J Orthod Dentofacial Orthop.

[5] Vermette ME, Kokich VG, Kennedy DB (1995). Uncovering labially impacted teeth: Apically positioned flap and closed-eruption techniques.. Angle Orthod.

[6] Czochrowska EM, Stenvik A, Album B, Zachrisson BU (2000). Autotransplantation of premolars to replace maxillary incisors: A comparison with natural incisors.. Am J Orthod Dentofacial Orthop.

[7] Lin YT (1999). Treatment of an impacted dilacerated maxillary central incisor.. Am J Orthod Dentofacial Orthop.

[8] Berglund L, Kurol J, Kvint S (1996). Orthodontic pre-treatment prior to autotransplantation of palatally impacted maxillary canines: Case reports on a new approach.. Eur J Orthod.

[9] Saad AY, Abdellatief ES (1996). Surgical repositioning of unerupted anterior teeth.. J Endod.

[10] Andresen JO (1992). Atlas of replantation and transplantation of teeth..

[11] Andreasen JO., Petersen JK., Laskin DM. (1997). Textbook and color atlas of tooth impactions..

[12] Azaz B, Steiman Z, Koyoumdjisky-Kaye E, Lewin-Epstein J (1980). The sequelae of surgical exposure of unerupted teeth.. J Oral Surg.

